# Does Public Awareness Matter to Achieve the UN's Sustainable Development Goal 6: Clean Water for Everyone?

**DOI:** 10.1155/2022/8445890

**Published:** 2022-10-06

**Authors:** Sohaib Mustafa, Khalid Jamil, Lifu Zhang, Mengisti Berihu Girmay

**Affiliations:** ^1^College of Economics and Management, Beijing University of Technology, 100124 Beijing, China; ^2^School of Economics and Management, North China Electric Power University, Beijing, China; ^3^Department of Translation, Lingnan University, Hong Kong, China; ^4^School of Information Science, Addis Ababa University, Ethiopia

## Abstract

United Nations set a Sustainable Development Goal to provide clean water for everyone (SDG 6). The successful implementation of SDG 6 is still miles to go. Public awareness's role as a key factor in achieving Sustainable Development Goal 6 is vital but received less attention from researchers in the past. To understand the role of public awareness and other relevant factors in achieving SDG 6, we have collected a cross-sectional dataset from a developing country and applied a partial least square structural equation modelling approach. The results revealed that willingness to pay for clean water, social influence, and facilities provided by the government, public awareness, and knowledge about contaminated water positively influence the households' intentions to use clean water. We also found that public awareness partially mediates relationships. Study results have useful policy implications for governments, NGOs, and other stakeholder organizations working on achieving SDG 6 in developing countries.

## 1. Introduction

The United Nations General Assembly in 2015 established 17 Sustainable Development Goals, one of which is “Ensure availability and sustainable management of water and sanitation for all.” The official phrase for this goal is “Ensure availability and sustainable management of water and sanitation for all [1].” Clean water and sanitation should be accessible to everyone everywhere, which is the focus of Sustainable Development Goal 6 (SDG 6 or Global Goal 6). There are eight subgoals that need to be accomplished by the year 2030. Eleven different indicators will be used to determine how much progress has been made towards the objectives [1].

Drinking water that is safe and affordable; ending open defecation and providing access to sanitation and hygiene; improving water quality, safe reuse, and wastewater treatment; increasing water-use efficiency and ensuring freshwater supplies; implementing integrated water resource management; and protecting and restoring water-related ecosystems are the six “outcome-oriented targets” that have been established. Expanding aid for clean water and sanitation infrastructure in developing nations and bolstering community participation in water and sanitation management are the two “ways of getting there” that are referred to as “means of attaining” goals [2].

In 2017, 2.2 billion people did not have access to drinking water managed properly, and 4.2 billion people did not have access to sanitation managed securely [3]. Three billion people around the globe do not have access to even the most basic handwashing facilities in their homes [3]. Around the globe, two out of every five healthcare facilities lack soap and water and alcohol-based hand rubs (2016) [3]. The COVID-19 pandemic has increased the significance of this aim in a significant way [4]. On the other hand, this epidemic might make it more difficult for water companies to achieve their goal by increasing the amount of income they lose, which is money that would normally be invested [5].

There is a strong connection between SDG 6 and the other Sustainable Development Goals (SDGs). For instance, making progress towards SDG 6 will enhance health (part of SDG 3) and increase school attendance, reducing poverty. In April 2020, António Guterres, the Secretary-General of the United Nations, made the following statement: “Today, Sustainable Development Goal 6 is badly off track.” He also stated that this “is hindering progress on the 2030 Agenda, the realization of human rights, and the achievement of peace and security around the world [6].”

Previous studies in this regard studied the threat to SDG 6 from urban drought [7], educational and citizen initiatives to support SDG 6 [8], assessing transformed urban agglomerations from the viewpoint of the water planetary boundary for SDG 6 [9], and water governance and SDG 6 achievability in India [10]. Still, ignoring the end-users' perspective that is the essential part of achieving SDG 6, none of the past studies focused on willingness to pay for clean water (WPCW), social influence (SI), facilitation provided by the government (FPG), knowledge about contaminated water (KCW), public awareness (PA), and intention to use clean water (IUCW). Neither have they explored the mediation role of public awareness in this context.

In addition to this, several studies have been conducted in countries where the education level and per capita income are stable, such as the BRICS Group: working towards actualization SDG 6 [11], UAE's commitments towards SDG 6 [12], but the successful achievement of UN's SDG 6 and factors influencing its implementation is unexplored in the countries where political instability, poverty, unemployment, education, and health are major hurdles in its way. We have selected Pakistan as a sample and collected a cross-sectional dataset to measure the influence of factors influencing the public intentions to use clean water. With this research gap, the following research questions are ones that we have suggested to answer in our study.

RQ1: How far do personal and economic factors and facilities provided by governments influence public intentions to use clean water?

RQ2: Does public awareness plays any role as a mediator in public intention to use clean water?

**Figure 1:**
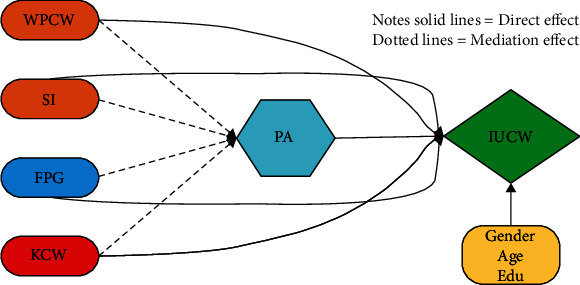
Conceptual framework. Note: WPCW: willingness to pay for clean water; SI: social influence; FPG: facilitations provided by the government; KCW: knowledge about contaminated water; PA: public awareness; IUCW: intention to use clean water.

To answer these research questions, we have proposed a model presented in Figure 1 and collected the cross-sectional dataset from urban and rural residents of Pakistan. We have used the partial least square structural equation modelling approach to conclude our results for the above-mentioned research questions. Results revealed that all five factors significantly influence Pakistani residents' intention to use clean water. In addition, public awareness partially mediates the understudy variables (WPCW, SI, KCW, and FPG) and the end-users' intention to use clean water. Policy-makers may use the study's findings to inform their efforts to accomplish Sustainable Development Goal 6, while researchers can use them to comprehend better the attitudes and actions of people living in developing nations with respect to this same United Nations initiative.

## 2. Literature Review and Hypothesis Development

To answer the proposed research questions, we have proposed a research model presented in Figure 1. In this model, we have incorporated the economic, personal, and social factors and facilities provided by governments to assess their influence on the public intention to use clean water. We got inspiration from the theory of planned behavior to propose the following model. It explains that “Intentions are assumed to capture the motivational factors that influence a behavior; they are indications of how hard people are willing to try, of how much of an effort they are planning to exert to perform the behavior. As a general rule, the stronger the intention to engage in a behavior, the more likely should be its performance” [13]. Hence, we believe that the willingness to pay for clean water (WPCW), social influence (SI), facilitation provided by the government (FPG), knowledge about contaminated water (KCW), and public awareness (PA) will influence public intentions to use clean water (IUCW) and be helpful in achieving UN's SDG 6.

### 2.1. Willingness to Pay for Clean Water

When it comes to deciding what to buy, customers are often persuaded in their decisions by a number of different economic concerns. It is well established that customers' income levels play a significant role in their choices on whether or not to make purchases [14, 15]. Paying for clean drinking water in the form of mineral water, tap water, and installing water filtration plants or water treatment plants are some common examples involving money, and it all depends on the household income [16, 17]. We expect that households' willingness to pay for clean water will positively influence their intention to use it. When individuals think about their health and associated health risks and compare them with the cost and possible reasons behind these health risks, they often go to pay for safe alternatives and adopt precautions. Hence, we proposed the following:

H1: Willingness to pay for clean water will positively influence household intention to use clean water, which will help achieve SDG 6.

### 2.2. Social Influence

People's perceptions of what others believe about the appropriateness of using a certain service, technology, or activity provide the foundation for forming normative beliefs [18–20]. When society decides whether or not to adopt emerging innovations, social influence plays a role. The research results indicate that it is an accurate predictor, yet there are circumstances in which it does not affect a person's decision. According to the findings of certain researchers, social factors are a significant factor in the adoption of environmentally friendly items [20], e-commerce [21], and 5G technology [22]. It has been shown that subjective standards significantly impact the amount of customer satisfaction that may be attained [17]. We hope that as a result of this, customers in developing nations will be motivated to adopt and use clean water, which will assist in the construction of a sustainable healthy society and prevent viral infections and eventually help in attaining SDG 6. Even while there is a lot of societal pressure on individuals to drink groundwater, it is almost always harmful, particularly in industrialized regions [16, 23, 24]. Hence, we expect that social influence will positively influence users' intention to use clean water and will be a key pillar in achieving SDG 6.

H2: Social influence will positively influence household intentions to use clean water and will be a helping hand in achieving SDG 6.

### 2.3. Facilitations Provided by the Government

To attain UN SDG 6 and provide basic facilities to the subjects, every government is providing facilities to its subjects. These facilities contain water supply, education, electricity and awareness or help in achieving a better lifestyle. The better infrastructure a government can provide its citizens directly influences their lifestyle [4, 24, 25]. The term “facilitating conditions” [5] is used to describe the methods and assets that are put into play in order to take advantage of a newly developed technology or product [20]. Hence, it is expected that governments and semigovernment institute initiatives and facilities will influence households' intentions to use clean water.

H3: Facilitations provided by the government will positively influence public intentions to use clean water that will be helpful in achieving SDG 6.

### 2.4. Knowledge about Contaminated Water

Human activity is responsible for polluting many of the world's water sources, including lakes, rivers, oceans, aquifers, and wells. This phenomenon is widespread across the world's waterways. Because of human intervention, water's physical, chemical, and biological properties have been altered, and the resulting water is toxic to all life forms. People who drink contaminated water or swim in filthy water run the risk of developing skin rashes, as well as cancer, reproductive disorders, typhoid fever, and stomach illnesses [26, 27]. People are more likely to avoid the usage of harmful products and prefer to use those products that can help them maintain good health and avoid health risks if they are informed about the benefits of a particular technology or product as well as the risks associated with using it, as this is the case when they have knowledge about the contaminated water and risks associated with its use. Knowledge about the advantages and related health risks is important in moulding human behavior towards modifying the human lifestyle and eating or drinking habits. This knowledge may either improve or harm a person's health. It has also been shown that providing individuals with environmental knowledge may enhance their views of environmental danger, environmental difficulties, and green purchasing patterns [18, 20, 22, 28]. Hence, we propose the following:

H4: Knowledge about contaminated water and the risks associated with its use will influence households to use clean water, and it will be a key determinant in achieving UN's SDG 6.

### 2.5. Public Awareness

A person's degree of awareness, which can be described as their grasp or acknowledgement of the advantages and downsides of the innovation, plays a significant role in determining whether or not they would accept an innovation, product, or activity in society [18, 22, 28]. A considerable percentage of individuals have a poor grasp of the benefits of utilizing clean water as a cure to prevent illnesses caused by polluted tap water, as shown by various research results. In the past, academics have seldom concentrated their study on investigating this aspect of customers' propensities to use clean water as their primary beverage [29, 30]. Researchers have claimed that users who are aware of some issues have the capacity to make better decisions compared to those without knowledge and awareness about the issues [18, 28]. Hence with this literature, we propose the following:

H5: Public awareness positively influences users' intentions to use clean water that will help achieve the UN's SDG 6.

### 2.6. Public Awareness as a Mediator

Public awareness is a strong predictor that plays a role in shaping consumers' behavior towards certain decision-making. We assume in our model that public awareness has also played its role as a mediator apart from the direct influence of the understudy variable. Previous studies have found that public awareness is influenced by age, education level, social status, recycling knowledge, public behavior, and willingness to participate in household waste treatment [31, 32]. Our understudy variable inserts their indirect influence with the mediation of public awareness; i.e., willingness to pay for clean water will increase public awareness. It will enhance the intention to use clean water. When consumers interact with each other, their awareness will also influence. The same is the case with facilitation provided by the government. As much as the government provides facilitations to facilitate citizens, it will enhance the awareness that leads to the intention to use clean water. Hence, we propose the following:

The relationship between WPCW (1a), SI (2a), FPG (3a), KCW (4a), and IUCW is mediated by public awareness.

## 3. Methodology

### 3.1. Data Collection

In order to acquire a dataset, we relied on a proven construct derived from earlier research. Table S2 in Supplementary Materials presents the detailed measurement items of the construct utilized to obtain the sample response. We have made some minor alterations to the phrasing of the measurement items in order to ensure that we get accurate responses and that they are the best fit for our study. The revised version of the construct was accepted by two academics in order to move forward with the study. First, we did some preliminary research in the form of a pilot study, and then, we moved on to the more extensive survey. For this aim, ten households and fifteen students at the master's level were chosen to assess the finalized questionnaire's readability and determine how long it took respondents to respond. The individuals who took part in the pilot study and the preliminary findings of the pilot study presented positive indicators that more research should be conducted [33]. Because of the potential for bias, the respondents from the pilot study were not included in the final sample.

We have decided to collect data through the use of an online survey so that we can eliminate the possibility of human error in the data handling process. We have segmented our population into two clusters depending on the literacy level, population concentration, and other facilities (rural and urban). Within these clusters, we used a method called systematic sampling to select one shopper from every ten who went to the supermarkets to do their grocery shopping. One of the most effective methods for obtaining responses from a diverse community is using this method [22, 34]. With the support of Google Forms, both the administration of the survey and the collection of responses were successfully carried out. In order to prevent having to make several attempts, for the purpose of data cleansing and to gather follow-up replies, respondents needed to enter their mobile phone numbers. The survey was carried out over two weeks, beginning in the third week of April 2022 and ending in the fourth week of the same month.

Before collecting any information or responses from any of the respondents, the researchers made sure to explain the goal of the study to each one of them and get their agreement. For the purpose of measuring the reaction, we have used a Likert scale of seven points, with “1 indicating strongly disagreeing and 7 as strongly agreeing.” According to the findings of the aforementioned research, the Likert scale with seven points is superior to higher-order alternative scales since it is more accurate and simpler to use [35]. A total of 600 questionnaires were dispersed. Four hundred twenty-three valid responses were collected for a response rate of 70.5%. The sample size is substantially larger than the minimum requirement of 10 times for each construct component in order to do statistical analysis [36].

### 3.2. Demographics of Respondents

To better comprehend our study sample and its characteristics, we have collated the participant's age, gender, education level, occupation, and residency status. Information about the demographics of our whole sample (423 people) is provided in Table 1.

### 3.3. Common Method Variance

The common method variance (CMV) approach is a method that can be utilized to mitigate the impact of the social desirability effect. If the data for the study came from a single source and if the first element accounted for more than forty percent of the total variation, then the CMV may be a major issue for any study [37]. In the current investigation, a single component analysis developed by Harman was utilized as a statistical technique to account for the likelihood of common method bias. The results of an exploratory analysis of factors using the principal axis factors approach revealed that a single factor accounted for just 31.05% of the variation across measurements. This figure is lower than the determined cut-off value, which was 50%.

Consequently, this demonstrates that the risk of CMV is reduced in this research. However, to provide further evidence in support of the process described above, another method, which controlled for the effects of a single unquantified latent approach component [37], was used. It was discovered that the measurement factor loading for the common latent component was 0.47, which indicates that the common factor accounted for just 27.04% of the variation across measurements. This score is below the criterion of 50%, indicating that the data are free from any potential biases of a subjective norm or shared variation among the variables examined.

### 3.4. PLS-SEM

PLS-SEM was the method we decided to go with since it is frequently suggested for use in research projects that aim to anticipate and examine the dependent variables to explain the most practical variation. As a result, we decided to utilize it for our research. As a consequence of this, the PLS-SEM technique is the most effective way of creating forecasts [20, 38]. In addition to this, it is able to deal with the measurement (outer) and structural (inner) models simultaneously. When employing the PLS-SEM method, it is feasible to get more precise conclusions while having a smaller sample size. As a consequence of this, it seems that the PLS-SEM approach is the most appropriate one for this investigation. Recent research has shown an increase in interest in making use of the PLS-SEM methodology due to the potential benefits it offers in the field of management science [28, 39].

PLS is more conducive to finding. Covariance-based SEM is more suited to testing and verification of well-established theories. The time to choose PLS is when your theories are still immature. As distinguishing between confirmatory and exploratory studies is not as easy as it may seem, this criterion requires further consideration. The consideration of data dispersion is another subject. Covariance-based SEM requires properly distributed data. PLS-SEM, on the other hand, makes no assumptions about the underlying data distributions. There is also the issue of sample size; covariance-based SEM studies need far more data than previously collected and analyzed. However, lower sample numbers are sufficient for PLS-SEM to converge.

PLS-SEM makes implementing formative measurement models simpler and more intuitive than covariance-based SEM. An additional factor is that PLS-SEM can simply and effectively manage increasingly complicated models. As such, PLS-SEM should be considered as the preferred option if formative measurement approaches are to be used.

In the process of PLS path modelling, the indicators of the constructs are evaluated in two different ways to ensure that they are reliable and accurate: (a) “the measurement model evaluation ensures the consistency and validity of the outer mode,” and (b) the structural model estimation helps to identify the inner model or connection among the latent components. These assessments are performed to ensure that the indicators are reliable and accurate [36].

### 3.5. Multivariate Assumptions

According to [15, 20], it is required to evaluate the multivariate assumptions of multicollinearity, homoscedasticity, and linearity before doing any multivariate testing. This must be done before performing any multivariate tests. During the survey phase, when the data was being collected, we ensured the respondent's anonymity and made it apparent that there was no correct or incorrect answer. We followed the lead of other researchers and used the Kolmogorov-Smirnov test to determine whether or not the data distribution was normal; however, the results indicated that it was not [40, 41]. In terms of linearity, the nonlinear and linear interactions between independent and dependent constructs are confirmed in Supplementary Materials (Table S3). In order to determine whether the model suffered from collinearity, the VIF scores were examined. According to [36], VIF values lower than 5 suggest that the data acquired does not include any problems related to collinearity. All of the indicators have VIF scores lower than 5, as shown by the outcomes of this study. Therefore, the fact that there is no collinearity issue with the dataset is evidence that the model is resilient.

As the last step, we generate a scatter plot of the regression normalized predicted value, and the residual value shows that the data are consistent with this hypothesis. This was accomplished by following the methodology of past research [14, 42]. The loadings, as well as the crossloadings of the indicators, may be found in Supplementary Materials (Table S1).

### 3.6. Measurement Model

**Figure 2:**
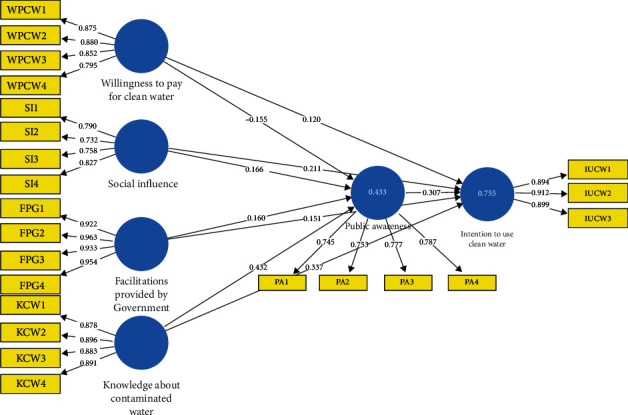
Measurement model.

According to the research that was conducted by [43], the reliability of a measurement model is determined by both its discriminant and convergent validity. Indicator loadings and Cronbach's alpha (*α*) were used in the analysis to determine the instrument's level of dependability. The indicators of the constructs were evaluated using convergent validity to see whether or not they were able to measure the variables under investigation accurately. When expressing the total variance in the indicators, Average variance extracted (AVE) is used, while composite reliability (CR) is utilized to demonstrate the dependability of the variables (Table 2). The model has component factor loadings of at least 0.6, which was the minimum required for inclusion (Figure 2). The assessed values of *α* are much higher than the cut-off value of 0.7, the CR for all variables is more than 0.7, and the AVE was discovered to be significantly greater than 0.50, a suggested number by specialists (Table 2). These findings provide evidence that the construct investigated in this research may be trusted [36, 43, 44].

In conclusion, the Fornell-Larcker criteria were used so that we could ascertain the discriminant validity of the research instrument before continuing to the next stage. It has been shown that a strong discriminant validity exists. The results of using the Fornell-Larcker criteria are shown in Table 3.

### 3.7. Structural Model Assessment

The PLS-SEM assessment procedure continues with the following stage, which is the structural model evaluation. Components of the structural path model assessment include assessing the predictive relevance of the model, the multicollinearity, the empirical significance of the path coefficients, and the degree of confidence in the results [21, 36, 43]. Following a predetermined protocol, the findings of this investigation were broken down and analyzed in order to draw conclusions. The *R*^2^ value of the first model (Table 4) for direct effect analysis on intention to use clean water is 0.755 (*Q*^2^ = 0.606), while *R*^2^ for the mediating variable public awareness is 0.433(*Q*^2^ = 0.348).

**Figure 3:**
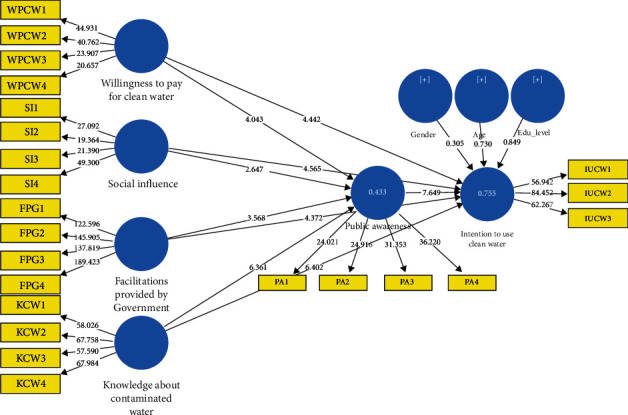
Path model.

We have run 5000 resamples of bootstrapping (Figure 3) by following the earlier researcher [21, 22]. The direct path results in model 1 revealed that all the independent variables positively influence the dependent variable with *p* value less than 0.001, i.e., PA -> IUCW (*β* = 0.312, *T* − value = 8.027, *p* value < 0.001), FPG -> IUCW (*β* = 0.148, *T* − value = 4.326, *p* value < 0.001), KCW -> IUCW (*β* = 0.338, *T* − value = 6.496, *p* value < 0.001), SI -> IUCW (*β* = 0.209, *T* − value = 4.603, *p* value < 0.001), WPCW -> IUCW (*β* = 0.12, and *T* − value = 4.427, *p* value < 0.001). In contrast, the control variables gender, age, and education were found insignificant in model 1 (Table 5). With these results, we have accepted hypotheses H1-H5.

### 3.8. Mediation Analysis

In addition to the direct path assessment in the model, we have run model 2 with the same bootstrapping sample and accessed the mediation effect of the public awareness (Table 6) between FPG, KCW, SI, and WPCW on IUCW. The model 2 analysis results revealed a decrease in the *β* value for direct relations. The specific indirect effects are as follows: KCW -> PA -> IUCW (*β* = 0.134, *T* − value = 4.959, *p* value < 0.001), WPCW -> PA -> IUCW (*β* = −0.048, *T* − value = 3.893, *p* value < 0.001), FPG -> PA -> IUCW (*β* = 0.05*T* − value = 3.253, *p* value = 0.001), and SI -> PA -> IUCW (*β* = 0.051, *T* − value = 2.437, *p* value = 0.015). Hence, the mediation results presented in Table 6 revealed a partial mediation of public awareness. The effect of understudying independent variables is passing through the mediator of public awareness. Public awareness as a mediator explains the independent variables' influence on the dependent variable.

With these results, we have accepted the hypotheses H1a-H4a.

## 4. Discussion

This study is conducted to understand the influential factors that influence the household intentions to use clean water and can be helpful in achieving the united nations Sustainable Development Goal 6. Based on the theory of planned behavior, we have proposed a model and collected a cross-sectional dataset from Pakistan, one of the underdeveloped countries facing economic, political, and infrastructural issues as hurdles in achieving UN Sustainable Development Goals. Our study access the economic, personal, and infrastructural barriers and households' perception of these factors and how these factors influence shaping their behavior in using clean water, which is necessary to avoid health risks.

We have presented two research questions to study the topic and hypotheses 1-5 to answer research question 1 and hypotheses 1a-4a to answer research question 2. H1-H5 access the direct effect, and H1a-H4a access the mediation effect of public awareness. We have used the PLS-SEM approach to conclude our results.

Results revealed that willingness to pay for clean water significantly positively influences the intention to use clean water (H1) and public awareness mediates the relationship (H1a), but the mediation magnitude is negative (competitive mediation). It means households who are willing to pay for clean water are inclined to use clean water. But if they are aware of the cost because of the low-income country, they hesitate to pay for it and need it as a complimentary from the government. Although the relationship in H1 is positive, its magnitude (*β* = 0.072) is minimal when a mediator of public awareness is exposed in the model. In mediation, public awareness further weakens the relationship influence. In addition, it implies that households in developing countries are less concerned about the awareness and possibly the cost factor to overcome the awareness and direct care of their health and associated health risks. However, if households are aware of the price and associated health risk and prevention costs, they are not conscious about caring about their health issues. Because they live in a low-income country and possibly have low income, they are hesitant to pay for clean water and expect the government to provide it free of charge. Social influence (H2) positively influences the IUCW, and it is also positively mediated by public awareness (H2a) (complementary mediation). The possible reason behind it can be that people are socially influenced; if someone from the community is concerned about their health and use clean water for drinking and sanitation, fellow community members will start following it. Furthermore, when community members interact, they argue about the benefits and drawbacks of using clean water and the associated health that increase public awareness among individuals and as a whole society that leading to the intention to use clean water [16, 18, 23].

FPG (H3) has a significant positive influence on IUCW, and this relationship is also mediated by PA (H3a). The possible reason behind this relationship is that government facilities influence citizens as it is the outcome of their direct and indirect taxes that they pay for necessities. Although this is one of the basic responsibilities of government to provide clean water to all citizens, international organizations such as the United Nations, World Bank, and Asian development bank provide funds to developing countries to meet the Sustainable Development Goal 6. Hence, infrastructure and other facilities provided by the government aligned with United Nations' Sustainable Development Goal 6 positively influence and increase public awareness about the benefits of using clean water.

Knowledge about contaminated water (H4) positively influences the intention to use clean water, and public awareness mediates (H4a) the relationship between KCW and IUCW. It means as much as an individual knows about the use of contaminated water and its hazardous outcomes or benefits using clean water, as much as they will be inclined to use clean water and avoid contaminated water. Knowledge about contaminated water is a direct component of public awareness; as much an individual has the knowledge, they will be aware of the consequences and vigilant. Hence, the mediation of public awareness between KCW and IUCW is obvious. It is consistent with the previous studies that knowledge about a certain issue enhances awareness [20, 22].

Public awareness (H5) has a significant factor behind the use of clean water and a significant mediator in the process. The possible justification is awareness urge human to act wisely and smartly and pick whatever is right for them. Hence, if we have to pursue Sustainable Development Goal 6, we must create public awareness and engage individual efforts to accomplish the goal on a large scale.

We also observed that the demographic factors incorporated as control variables, gender, age, and education, have no substantial influence on our study. It contradicts the results of the previous studies that claim age and education are determinantal factors in shaping household awareness and behavior [31, 32].

## 5. Policy Implications

The job of the government and nongovernmental organizations is to build large-scale awareness campaigns on the need for good hygiene, including the points that water that seems clean may still be dangerous and that there is a requirement for household water treatment. Instead of using health concerns as a justification to get families to buy a filter, try appealing to their aspirations, using social stigma, and building trust. In addition, the government should make it possible for families unable to pay the whole cost upfront to make payments using their mobile phones or microcredit. The government and nongovernmental organizations can provide one-time financial assistance to low-income families. Filters, whether they are free or subsidized, should not disrupt markets but rather assist supply chains. One option is to provide free vouchers, which a family may use to “purchase” a filter from a retailer of their choosing. It is vital for governments and nongovernmental organizations [45] and the corporate sector to collaborate in order to scale up household water treatment and safe storage, and it is also essential that regulations be in place.

We also suggest considering building a piped infrastructure with home connections rather than community water tap points in communities with at least 500 people since this will eliminate the need for individuals to walk to get the water supply. Consumers are generally willing to pay for a service, such as water delivery to their homes, even if it costs more. Keeping in view the income level of rural and urban areas, it may be possible to slightly raise the price of water in metropolitan areas, and the additional revenue might then be used to support water provision in rural regions. People should be given support to put adequate infrastructure in areas where the cost of providing water access per person is very high because there are no low-cost options available (places with extensive water layers or very rough terrain).

In general, for developing countries and specifically for Pakistan, UN has its setup with the name of UNDP (Pakistan). This setup organized by UN actively participates in developing nations and helps to achieve sustainable growth and UN goals set for 2030. A model of 80-20 public UNDP partnerships can help in establishing a well-organized structure to achieve SDG6. We suggest UN make UNDP more deep routed and provide clean water facilities with the cooperation of local communities rather than providing funds to NGOs and other local bodies. We strongly encourage the involvement of local community organizations and social communities in providing clean water in underdeveloped areas such as Thar in Sindh and the South Punjab region.

## 6. Limitations

We tried to overcome the possible limitation by implying procedural and statistical instruments; still, our study lacked in some areas. These limits can be used as a possible future research avenue for researchers. Firstly, we only gathered data from one nation for our sample; a potential selection bias affects our findings' generalizability. Researchers are strongly urged to confirm the findings by researching in many countries simultaneously. Researchers can compare developed nations with underdeveloped nations and underdeveloped nations with others by distinguishing the geographic borders of the countries being compared, for example, Asian countries and African countries. Second, we did not assess the household earnings in our sample population. The degree of a consumer's income may have an impact on the process, and the priorities of customers might shift depending on their level of money. In further research, we recommend including the impact of one's income level. Thirdly, as a control variable, we look at the level of education. Future scholars will be able to investigate the profound impact of educational levels, in which the literate and uneducated members of the community can be crosscompared, and offer strategy statements to educate the masses about ecological problems and encourage people to use clean water to avoid health risks.

## 7. Conclusion

In response to the results revealed from our work, we concluded that public awareness about the use of clean water among developing countries' residents is limited. It needs to improve if the successful implementation of Sustainable Development Goal 6 needs to be achieved by 2030. We have also found that if households have awareness about the use of clean water and the risk associated with the use of contaminated water, the influence of willingness to pay for clean water, facilities provided by the government, social influence, and knowledge about contaminated water is more when they do not have any awareness. Hence, public awareness is a determinantal factor in achieving United Nations SDG 6. Our study results are also helpful in understanding the barriers to implementing SDG 6 in developing countries where infrastructure and economic and political instability are hurdles in development.

## Figures and Tables

**Table 1:** Demographic characteristics.

Characteristics	Range	Frequency	Percentage
Gender	Male	241	57%
	Female	182	43%
Age	18-25 year	109	25.8%
	26-35 year	147	34.8%
	36-45 year	115	27.2%
	>45 year	52	12.3%
Education	High school or less	31	7.3%
	Bachelor	151	35.7%
	Master	240	56.7%
	Doctorate	1	0.20%
Occupation	Student	106	25.1%
	Govt. employee	96	22.7%
	Private company employee	132	31.2%
	Businessman/women/other	89	21.0%
Residential status	Urban	297	70.2%
	Rural	126	29.8%
Access to clean water	Yes	333	78.7%
	No	90	21.3%

**Table 2:** Reliability and validity analysis.

Constructs	Items	Loadings	T statistics	VIF	*α*	CR	AVE
Facilitations provided by government	FPG1	0.922^∗∗∗^	122.519	2.818	0.953	0.966	0.877
	FPG2	0.936^∗∗∗^	148.248	2.978			
	FPG3	0.933^∗∗∗^	138.331	2.902			
	FPG4	0.954^∗∗∗^	191.423	3.77			
Intention to use clean water	IUCW1	0.894^∗∗∗^	56.443	2.305	0.885	0.929	0.813
	IUCW2	0.912^∗∗∗^	84.802	2.776			
	IUCW3	0.899^∗∗∗^	62.512	2.593			
Knowledge about contaminated water	KCW1	0.878^∗∗∗^	59.981	2.633	0.91	0.937	0.787
	KCW2	0.896^∗∗∗^	68.147	2.916			
	KCW3	0.883^∗∗∗^	59.238	2.778			
	KCW4	0.891^∗∗∗^	67.704	2.807			
Public awareness	PA1	0.745^∗∗∗^	24.148	1.437	0.765	0.85	0.587
	PA2	0.753^∗∗∗^	25.23	1.447			
	PA3	0.777^∗∗∗^	32.338	1.553			
	PA4	0.787^∗∗∗^	36.329	1.498			
Social influence	SI1	0.790^∗∗∗^	26.822	2.08	0.785	0.859	0.605
	SI2	0.732^∗∗∗^	19.014	1.906			
	SI3	0.758^∗∗∗^	21.753	1.518			
	SI4	0.827^∗∗∗^	47.967	1.688			
Willingness to pay for clean water	WPCW1	0.875^∗∗∗^	44.132	2.498	0.873	0.913	0.725
	WPCW2	0.880^∗∗∗^	40.609	2.667			
	WPCW3	0.852^∗∗∗^	24.497	2.438			
	WPCW4	0.795^∗∗∗^	20.505	1.884			

Notes: *α* > 0.7; CR > 0.7; AVE > 0.5; VIF < 5; ^∗∗∗^significant at *p* < 0.001.

**Table 3:** Fornell-Larcker's criteria (discriminant validity).

	Mean	Std. dev	FPG	IUCW	KCW	PA	SI	WPCW
FPG	5.15	1.34	0.936					
IUCW	4.94	1.44	0.599	0.902				
KCW	4.88	1.32	0.57	0.799	0.887			
PA	4.60	1.39	0.461	0.698	0.617	0.766		
SI	4.52	1.34	0.462	0.724	0.762	0.545	0.778	
WPCW	4.35	0.97	0.148	0.246	0.215	-0.011	0.162	0.851

Note: WPCW: willingness to pay for clean water; SI: social influence; FPG: facilitations provided by the government; KCW: knowledge about contaminated water; PA: public awareness; IUCW: intention to use clean water.

**Table 4:** Coefficient determination and blindfolding results.

Exogenous constructs	Overall model	
	*R* ^2^	*Q* ^2^
IUCW	0.755	0.606
PA	0.433	0.348

**Table 5:** Direct paths.

Paths	Effects	Model 1			
		*β*	SD	*T*-value	*p* value
WPCW -> IUCW	(H1)+	0.12	0.027	4.427	^∗∗∗^
SI -> IUCW	(H2)+	0.209	0.045	4.603	^∗∗∗^
FPG -> IUCW	(H3)+	0.148	0.034	4.326	^∗∗∗^
KCW -> IUCW	(H4)+	0.338	0.052	6.496	^∗∗∗^
PA -> IUCW	(H5)+	0.312	0.039	8.027	^∗∗∗^
*Control variables*					
Gender -> IUCW		0.007	0.024	0.305	0.76
Age -> IUCW		-0.017	0.023	0.752	0.452
Education -> IUCW		0.021	0.026	0.839	0.402

Note: ^∗∗∗^significance at level *p* ≤ 0.001.

**Table 6:** Mediation analysis.

Paths	Effects	Model 2			
		*β*	SD	*T*-value	*p* value
WPCW -> IUCW	(H1)+	0.072	0.026	2.762	0.006
SI -> IUCW	(H2)+	0.260	0.049	5.293	^∗∗∗^
FPG -> IUCW	(H3)+	0.198	0.036	5.500	^∗∗∗^
KCW -> IUCW	(H4)+	0.473	0.056	8.520	^∗∗∗^
WPCW -> PA -> IUCW	(H1a)-	-0.048	0.012	3.893	^∗∗∗^
SI -> PA -> IUCW	(H2a)+	0.051	0.021	2.437	0.015
FPG -> PA -> IUCW	(H3a)+	0.05	0.015	3.253	^∗∗∗^
KCW -> PA -> IUCW	(H4a)+	0.134	0.027	4.959	^∗∗∗^

Note: ^∗∗∗^significance at level *p* ≤ 0.001.

## Data Availability

The dataset used in the study is available from the corresponding author at a reasonable demand.

## Supplementary Materials

Supplementary Materials Further details of crossloading, measurement construct, and correlation among variables can be found in Supplementary Materials as Table S1, Table S2, and Table S3.

## References

[1] Nation U., UN (2017). *Resolution adopted by the General Assembly on 6 July 2017, in A/RES/71/313*.

[2] Nation U., UN (2018). *Sustainable Development Goal 6- Synthesis Report 2018 on Water and Sanitation*.

[3] UN (2022). *Goals 6: Ensure availability and sustainable management of water and sanitation for all*.

[4] UNESC (2020). *Progress towards the Sustainable Development Goals*.

[5] IFC (2022). *The Impact of COVID-19 on the Water and Sanitation Sector*.

[6] Blazhevska V. (2022). *United Nations launches framework to speed up progress on water and sanitation goal*.

[7] Zhang X., Chen N., Sheng H. (2019). Urban drought challenge to 2030 sustainable development goals. *Science of the Total Environment*.

[8] de Lázaro Torres M. L., Borderías Uribeondo P., Morales Yago F. J. (2020). Citizen and educational initiatives to support Sustainable Development Goal 6: Clean water and sanitation for all. *Sustainability*.

[9] Yang Y., Cheng Y. (2021). Evaluating the ability of transformed urban agglomerations to achieve Sustainable Development Goal 6 from the perspective of the water planetary boundary: evidence from Guanzhong in China. *Journal of Cleaner Production*.

[10] Lindamood D. (2018). *Towards a more sustainable water future: water governance and Sustainable Development Goal 6 achievability in India, in UWSpace*.

[11] Filho W. L., Azul A. M., Brandli L., Wall T. (2021). BRICS consortium: toward implementing Sustainable Development Goal 6. *Partnerships for the Goals*.

[12] Umar T., Egbu C., Ofori G. (2020). UAE’s commitment towards UN Sustainable Development Goals. *Sustainable Development Goals*.

[13] Ajzen I. (1991). The theory of planned behavior. *Organizational Behavior and Human Decision Processes*.

[14] Mustafa S., Sohail M. T., Alroobaea R. (2022). Éclaircissement to understand consumers’ decision-making psyche and gender effects, a fuzzy set qualitative comparative analysis. *Frontiers in Psychology*.

[15] Mustafa S., Zhang W., Li R. (2021). Does environmental awareness play a role in E.V. adoption? A value-based adoption model analysis with SEM-ANN approach. *IEEE/WIC/ACM International Conference on Web Intelligence and Intelligent Agent Technology*.

[16] Sohaila M. T., Mahfoozb Y., Aftabc R., Yend Y., Talibe M. A., Rasoolf A. (2020). Water quality and health risk of public drinking water sources: a study of filtration plants installed in Rawalpindi and Islamabad, Pakistan. *Desalination and Water Treatment*.

[17] Shahab A., Shihua Q., Rashid A., Hasan F., Sohail M. (2016). Evaluation of water quality for drinking and agricultural suitability in the lower Indus plain in Sindh Province, Pakistan. *Polish Journal of Environmental Studies*.

[18] Sohail M. T., Elkaeed E. B., Irfan M., Acevedo-Duque Á., Mustafa S. (2022). Determining farmers’ awareness about climate change mitigation and wastewater irrigation: A pathway toward green and sustainable development. *Frontiers in Environmental Science*.

[19] Wang Z., Sohail M. T. (2022). Short- and long-run influence of education on subjective well-being: the role of information and communication Technology in China. *Frontiers in Psychology*.

[20] Mustafa S., Hao T., Jamil K., Qiao Y., Nawaz M. (2022). Role of eco-friendly products in the revival of developing countries' economies and achieving a sustainable green economy. *Science*.

[21] Mustafa S., Hao T., Qiao Y., Kifayat Shah S., Sun R. (2022). How a successful implementation and sustainable growth of e-commerce can be achieved in developing countries; a pathway towards green economy. *Science*.

[22] Mustafa S., Zhang W., Shehzad M. U., Anwar A., Rubakula G. (2022). Does health consciousness matter to adopt new technology? An integrated model of UTAUT2 with SEM-fsQCA approach. *Frontiers in Psychology*.

[23] Sohail M. T., Aftab R., Mahfooz Y. (2019). Estimation of water quality, management and risk assessment in Khyber-Pakhtunkhwa and Gilgit Baltistan Pakistan. *Desalination and Water Treatment*.

[24] Sohail M. T., Lin X., Lizhi L. (2021). Farmers’ awareness about impacts of reusing wastewater, risk perception and adaptation to climate change in Faisalabad District, Pakistan. *Polish Journal of Environmental Studies*.

[25] Sohail M. T., Ullah S., Majeed M. T., Usman A., Andlib Z. (2021). The shadow economy in South Asia: dynamic effects on clean energy consumption and environmental pollution. *Environmental Science and Pollution Research*.

[26] Sohail M. T., Ehsan M., Riaz S., Elkaeed E. B., Awwad N. S., Ibrahium H. A. (2022). Investigating the drinking water quality and associated health risks in metropolis area of Pakistan. *Frontiers in Materials*.

[27] Mahfooz Y., Yasar A., Guijian L. (2020). An assessment of wastewater pollution, treatment efficiency and management in a semi-arid urban area of Pakistan. *Desalination and Water Treatment*.

[28] Sohail M. T., Mustafa S., Ali M. M., Riaz S. (2022). Agricultural communities’ risk assessment and the effects of climate change: a pathway toward green productivity and sustainable development. *Science*.

[29] Zhao W., Chang M., Yu L., Sohail M. T. (2022). Health and human wellbeing in China: do environmental issues and social change matter?. *Frontiers in psychology*.

[30] Mahfooz Y., Yasar A., Sohail M. T. (2019). Investigating the drinking and surface water quality and associated health risks in a semi-arid multi-industrial metropolis (Faisalabad), Pakistan. *Environmental Science and Pollution Research*.

[31] Wang H., Liu X., Wang N. (2020). Key factors influencing public awareness of household solid waste recycling in urban areas of China: a case study. *Resources, Conservation and Recycling*.

[32] Han Z., Duan Q., Fei Y. (2018). Factors that influence public awareness of domestic waste characteristics and management in rural areas. *Integrated environmental assessment and management*.

[33] Kost R. G., de Rosa J. C. (2018). Impact of survey length and compensation on validity, reliability, and sample characteristics for ultrashort-, short-, and long-research participant perception surveys. *Journal of clinical and translational science*.

[34] Sekaran U., Bougie R. (2019). *Sampling, in research methods for business: a skill building approach*.

[35] Finstad K. (2010). Response interpolation and scale sensitivity: evidence against 5-point scales. *Journal of Usability Studies*.

[36] Hair J. F., Howard M. C., Nitzl C. (2020). Assessing measurement model quality in PLS-SEM using confirmatory composite analysis. *Journal of Business Research*.

[37] Podsakoff P. M., MacKenzie S. B., Podsakoff N. P. (2012). Sources of method bias in social science research and recommendations on how to control it. *Annual Review of Psychology*.

[38] Roldán J., Sánchez-Franco M. J. (2012). Variance-based structural equation modeling: Guidelines for using partial least squares in information systems research, in research methodologies, innovations and philosophies in software systems engineering and information systems. *IGI Global*.

[39] Tang Z., Shah S. K., Ahmad M., Mustafa S. (2022). Modeling consumer’s switching intentions regarding 5G Technology in China. *International Journal of Innovation and Technology Management*.

[40] Mustafa S., Zhang W., Naveed M. M. (2022). What motivates online community contributors to contribute consistently? A case study on stackoverflow netizens. *Current Psychology*.

[41] Awan F. H., Dunnan L., Jamil K. (2021). Mediating role of green supply chain management between lean manufacturing practices and Sustainable performance. *Frontiers in Psychology*.

[42] Mustafa S., Wen Z. (2022). How to achieve maximum participation of users in technical versus non-technical online Q&A communities?. *International Journal of Electronic Commerce*.

[43] Hair J. F., Risher J. J., Sarstedt M., Ringle C. M. (2019). When to use and how to report the results of PLS-SEM. *European Business Review*.

[44] Mustafa S., Qiao Y., Yan X., Anwar A., Hao T., Rana S. (2022). Digital students' satisfaction with and intention to use online teaching modes, role of big five personality traits. *Frontiers in Psychology*.

[45] Jahangoshai Rezaee M., Yousefi S., Hayati J. (2019). Root barriers management in development of renewable energy resources in Iran: an interpretative structural modeling approach. *Energy Policy*.

