# Supplementary material for: Does Public Awareness Matter to Achieve the UN's Sustainable Development Goal 6: Clean Water for Everyone?
Source: J Environ Public Health. 2022 Oct 6;2022:8445890. doi: 10.1155/2022/8445890 (PMC9560826; doi:10.1155/2022/8445890)
Supplement: Supplementary Materials — Further details of crossloading, measurement construct, and correlation among variables can be found in Supplementary Materials as Table S1, Table S2, and Table S3. [file 8445890.f1.docx]

**Table 1. Cross loadings**

|  | Facilitations Provided by Government | Intention to Use clean water | Knowledge about Contaminated Water | Public Awareness | Social Influence | Willingness to Pay for Clean Water |
| --- | --- | --- | --- | --- | --- | --- |
| FPG1 | **0.922** | 0.581 | 0.566 | 0.45 | 0.465 | 0.168 |
| FPG2 | **0.936** | 0.574 | 0.527 | 0.436 | 0.418 | 0.164 |
| FPG3 | **0.933** | 0.534 | 0.512 | 0.411 | 0.412 | 0.071 |
| FPG4 | **0.954** | 0.553 | 0.528 | 0.427 | 0.432 | 0.145 |
| IUCW1 | 0.553 | **0.894** | 0.752 | 0.673 | 0.639 | 0.255 |
| IUCW2 | 0.541 | **0.912** | 0.708 | 0.643 | 0.677 | 0.167 |
| IUCW3 | 0.526 | **0.899** | 0.698 | 0.566 | 0.641 | 0.243 |
| KCW1 | 0.482 | 0.707 | **0.878** | 0.537 | 0.629 | 0.245 |
| KCW2 | 0.549 | 0.727 | **0.896** | 0.555 | 0.697 | 0.189 |
| KCW3 | 0.435 | 0.653 | **0.883** | 0.527 | 0.686 | 0.171 |
| KCW4 | 0.551 | 0.742 | **0.891** | 0.569 | 0.692 | 0.16 |
| PA1 | 0.339 | 0.475 | 0.466 | **0.745** | 0.415 | 0.073 |
| PA2 | 0.377 | 0.498 | 0.468 | **0.753** | 0.39 | -0.046 |
| PA3 | 0.332 | 0.545 | 0.456 | **0.777** | 0.355 | -0.02 |
| PA4 | 0.363 | 0.609 | 0.499 | **0.787** | 0.498 | -0.036 |
| SI1 | 0.335 | 0.519 | 0.495 | 0.397 | **0.79** | 0.101 |
| SI2 | 0.362 | 0.43 | 0.499 | 0.331 | **0.732** | 0.12 |
| SI3 | 0.282 | 0.59 | 0.626 | 0.41 | **0.758** | 0.165 |
| SI4 | 0.447 | 0.667 | 0.709 | 0.522 | **0.827** | 0.118 |
| WPCW1 | 0.106 | 0.233 | 0.209 | -0.019 | 0.143 | **0.875** |
| WPCW2 | 0.104 | 0.224 | 0.202 | 0.014 | 0.153 | **0.88** |
| WPCW3 | 0.163 | 0.169 | 0.138 | -0.048 | 0.114 | **0.852** |
| WPCW4 | 0.141 | 0.2 | 0.171 | 0.008 | 0.136 | **0.795** |

**Table 2. Measurement Construct**

| **Factor** | **Construct** | | **Reference** |
| --- | --- | --- | --- |
| Facilitations Provided by Government | FPG1 | I have access to the water filtration plants installed by the government. | [1] |
|  | FPG2 | The government provided all necessary products and equipment required to access clean water. |  |
|  | FPG3 | Fast and free home delivery of clean water is available in my area. |  |
|  | FPG4 | I can communicate with the authorities if I have any issues with clean water. |  |
| Intention to Use clean water | IUCW1 | I intend to continue using clean water in the future. | [1] |
|  | IUCW2 | I plan to continue to use clean water frequently. |  |
|  | IUCW3 | I will always try to use clean water in my daily life |  |
| Knowledge about Contaminated Water | KCW1 | I am very knowledgeable about contaminated water and its related environmental and health issues. | [1] |
|  | KCW2 | I know that I adopt contaminated water. |  |
|  | KCW3 | I know that I buy will buy clean water as I am more cautious about my health. |  |
|  | KCW4 | Contaminated water is a serious threat to society and human health. |  |
| Public Awareness | PA1 | I am aware that the use of contaminated water is hazardous. | [2] |
|  | PA2 | I am aware that clean water can avoid several health diseases. |  |
|  | PA3 | I often try to read about the benefits of clean water. |  |
|  | PA4 | I am aware of the fact that it's the collective duty of society members to participate in the collective activities that improve our health. |  |
| Social Influence | SI1 | Society members who are influential to me think that I must use clean water. | [3] |
|  | SI2 | Society members who influence my behaviour think I must use clean water. |  |
|  | SI3 | Society members whose opinions I value prefer that I use clean water. |  |
|  | SI4 | I am inspired by society members who use clean water. |  |
| Willingness to Pay for Clean Water | WPCW1 | I am willing to pay for clean water. | [1, 3] |
|  | WPCW2 | The clean water supply price is reasonable in my area. |  |
|  | WPCW3 | I am willing to pay the society bill for clean water. |  |
|  | WPCW4 | I think everyone should pay a clean water bill. |  |

**Table 3. Correlations**

|  | | ***FPG*** | ***PA*** | ***WPCW*** | ***SI*** | ***KCW*** | ***IUCW*** |
| --- | --- | --- | --- | --- | --- | --- | --- |
| FPG | Pearson Correlation | 1 | .161^**^ | .149^**^ | .157^**^ | .209^**^ | .241^**^ |
|  | Sig. (2-tailed) |  | .001 | .002 | .001 | .000 | .000 |
|  | N | 423 | 423 | 422 | 423 | 423 | 423 |
| PA | Pearson Correlation | .161^**^ | 1 | .456^**^ | .456^**^ | .743^**^ | .708^**^ |
|  | Sig. (2-tailed) | .001 |  | .000 | .000 | .000 | .000 |
|  | N | 423 | 423 | 422 | 423 | 423 | 423 |
| WPCW | Pearson Correlation | .149^**^ | .456^**^ | 1 | .684^**^ | .605^**^ | .598^**^ |
|  | Sig. (2-tailed) | .002 | .000 |  | .000 | .000 | .000 |
|  | N | 422 | 422 | 422 | 422 | 422 | 422 |
| SI | Pearson Correlation | .157^**^ | .456^**^ | .684^**^ | 1 | .524^**^ | .506^**^ |
|  | Sig. (2-tailed) | .001 | .000 | .000 |  | .000 | .000 |
|  | N | 423 | 423 | 422 | 423 | 423 | 423 |
| KCW | Pearson Correlation | .209^**^ | .743^**^ | .605^**^ | .524^**^ | 1 | .813^**^ |
|  | Sig. (2-tailed) | .000 | .000 | .000 | .000 |  | .000 |
|  | N | 423 | 423 | 422 | 423 | 423 | 423 |
| IUCW | Pearson Correlation | .241^**^ | .708^**^ | .598^**^ | .506^**^ | .813^**^ | 1 |
|  | Sig. (2-tailed) | .000 | .000 | .000 | .000 | .000 |  |
|  | N | 423 | 423 | 422 | 423 | 423 | 423 |
| **. Correlation is significant at the 0.01 level (2-tailed). | | | | | | | |

References:

1. Mustafa, S., et al., *Role of eco-friendly products in the revival of developing countries' economies & achieving a sustainable green economy.* Frontiers in Environmental Science, 2022. **10** DOI: 10.3389/fenvs.2022.955245.

2. Sohail, M.T., et al., *Determining Farmers' Awareness About Climate Change Mitigation and Wastewater Irrigation: A Pathway Toward Green and Sustainable Development.* Frontiers in - Environmental Science, 2022. **10** DOI: 10.3389/fenvs.2022.900193.

3. Mustafa, S., et al., *How a successful implementation and sustainable growth of e-commerce can be achieved in developing countries; a pathway towards green economy.* Frontiers in Environmental Science, 2022. **10** DOI: 10.3389/fenvs.2022.940659.
